# Agarose‐Based Films Enriched With Ascorbic Acid for Inhibiting Enzymatic Browning and Extending the Shelf Life of Fresh‐Cut Apples

**DOI:** 10.1002/fsn3.72022

**Published:** 2026-06-11

**Authors:** Songul Bayrak, Hasan Ozdemir

**Affiliations:** ^1^ Department of Chemistry, Faculty of Science Ataturk University Erzurum Turkey

**Keywords:** agarose film, antimicrobial activity, antioxidant activity, ascorbic acid, enzymatic browning, PPO inhibition

## Abstract

In this study, agarose‐based films enriched with ascorbic acid (AA@Ag) were developed and evaluated for their effectiveness in reducing enzymatic browning in fresh‐cut apples. Structural and physicochemical analyses confirmed the successful incorporation of ascorbic acid (AA) into the agarose matrix, accompanied by concentration‐dependent modifications in film morphology and properties. The AA@Ag‐4 film exhibited reduced moisture content (6.70%), solubility (18.33%), and swelling ratio (39.40%), along with increased opacity (50.96%) compared to the pure agarose film. In antioxidant assays, AA@Ag‐4 exhibited high radical‐scavenging activity, with DPPH and ABTS values of 90.53% and 94.03%, respectively. Antimicrobial activity increased with increasing AA concentration, with inhibition zones reaching 30.6 mm against *Escherichia coli* (*E. coli)* and 30.0 mm against *Staphylococcus aureus (S.* *aureus* *)*. During 12 days of storage, coated apple samples maintained higher L* values and exhibited a lower browning index (BI) than the control group. Polyphenol oxidase (PPO) activity was also reduced, with AA@Ag‐4 samples showing 79.2 U/mL compared to 131.3 U/mL in the control. These findings demonstrate that AA@Ag films reduced enzymatic browning in fresh‐cut apples under the tested storage conditions, as evidenced by improved color stability and lower PPO activity. The observed antioxidant and antimicrobial properties indicate the potential of these films for active food packaging applications; however, further validation under real food storage conditions is required.

## Graphical abstract

To prevent enzymatic browning and quality loss in freshly cut apples, ascorbic acid‐enriched agarose films have been developed as biodegradable active packaging materials. Structural analyses confirmed that ascorbic acid was successfully incorporated into the polymer matrix, yielding compact, homogeneous films with improved physicochemical properties.

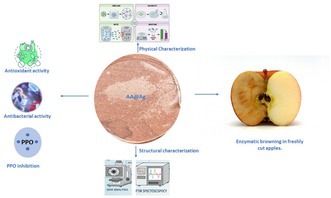

## 1 Introduction

Consumer interest in fresh‐cut fruits and vegetables has increased substantially because of their convenience and perceived nutritional value. However, mechanical operations such as peeling, slicing, and cutting disrupt cellular integrity, accelerate oxidative reactions, and stimulate enzymatic activity, particularly PPO‐mediated processes, resulting in rapid quality deterioration during storage. These reactions are generally associated with undesirable quality changes, including discoloration, texture deterioration, flavor alterations, and nutritional losses (Martínez et al. 2023; Oms‐Oliu et al. 2007). Various physical, chemical, and biological strategies have been reported in the literature to address these challenges. Prevention of browning by various remedies, including ozone treatment, electrolyzed water, amino acids, organic acids, natural plant extracts, and bioactive coatings, has also been reported (Ali et al. 2015).

It is important to distinguish between different preservation outcomes, which are often used interchangeably in the literature but represent fundamentally different quality parameters (Soliva‐Fortuny and Martin‐Belloso 2003; Sui et al. 2023). Antibrowning efficacy, primarily associated with inhibiting PPO activity, primarily affects visual appearance and consumer acceptance (Ali et al. 2025). In contrast, nutritional preservation relates to the retention of bioactive compounds such as phenolics and vitamins, whereas shelf‐life extension depends on a combination of microbial stability, physicochemical integrity, and sensory attributes, including texture and flavor (Ahmed et al. 2022; Sánchez‐González et al. 2011). Furthermore, commercial applicability requires not only functional performance but also cost‐effectiveness, scalability, and regulatory acceptance. In addition to postharvest treatments, it should be recognized that the intrinsic quality and shelf‐life potential of fresh‐cut produce are partially established during preharvest stages. Factors such as cultivation conditions, environmental stress, genotype, and maturity at harvest can significantly influence the initial biochemical composition of plant tissues, including phenolic content, antioxidant capacity, and enzymatic activity. These parameters, in turn, affect susceptibility to oxidative reactions and enzymatic browning during storage. Therefore, postharvest performance should be interpreted in conjunction with preharvest background rather than being attributed solely to coating or treatment effects (Fanourakis et al. 2025).

For example, previous studies have reported inconsistent antibrowning outcomes depending on the treatment type and product matrix; while Wills and Li demonstrated that arginine inhibited browning in fresh‐cut apples and lettuce, Zhao et al. reported contrasting results using plant extracts in potato systems. However, some of these approaches may have limitations related to cost, efficiency, or consumer acceptance, depending on the treatment conditions and the food matrix. Consequently, there remains a need to develop safer, more environmentally friendly, and user‐friendly alternatives (Ma et al. 2024; Wills and Li 2016; Zhao et al. 2020).

In recent years, the use of biopolymer‐based films and edible coatings has become increasingly important in food packaging technology. Biopolymers are considered promising alternatives to synthetic packaging materials due to their biocompatibility, biodegradability, and renewability. However, despite their advantages, film‐ and coating‐based preservation systems are not without limitations. For instance, such materials may alter gas‐exchange properties, potentially leading to anaerobic conditions or undesirable metabolic responses in fresh‐cut products. In addition, changes in optical properties, including increased opacity or surface gloss, may affect consumer perception and product acceptance. From a practical perspective, issues related to coating uniformity, scalability, processing conditions, and cost‐effectiveness may further limit their industrial applicability. Therefore, while biopolymer‐based coatings offer promising functional benefits, their performance and feasibility must be critically evaluated within a broader technological and commercial context (Ahmed et al. 2022; Falguera et al. 2011; Galus and Kadzińska 2015; Ma et al. 2024; Sánchez‐González et al. 2011).

Among biopolymers, polysaccharide‐based materials such as agarose (Ag) have received particular attention due to their strong gelling properties, optical transparency, and mechanical strength (Bilal et al. 2019). However, pure agarose films exhibit limited functionality and therefore require modification or enrichment with active compounds.

Natural antioxidants represent an effective strategy for enhancing film functionality, as they can scavenge free radicals and suppress oxidative reactions, thereby preserving the nutritional and sensory properties of foods. In addition, their incorporation into polymer matrices may enhance antimicrobial performance and storage stability (Deshmukh and Gaikwad 2024; Gulcin 2020). Therefore, the addition of natural antioxidants to biopolymer films is a promising approach to extend shelf life and improve food safety.

AA has attracted considerable attention in food preservation systems because of its strong reducing properties and compatibility with biopolymer‐based matrices (Pizzocaro et al. 1993; Suttirak and Manurakchinakorn 2010). It can delay lipid oxidation and enzymatic browning by scavenging reactive oxygen species and inhibiting PPO activity. Previous studies have shown that AA‐containing films based on polymers such as chitosan or PVA can effectively reduce browning and enhance antioxidant capacity in products such as mushrooms, grapes, and apples (Lo'Ay and Dawood 2017; Ojeda et al. 2019). However, the incorporation of AA into agarose‐based films and the relationship between agarose matrix behavior and multifunctional preservation performance in fresh‐cut systems have not been sufficiently explored. To address this research gap, agarose films (AA@Ag) with varying AA concentrations were developed and systematically evaluated for their structural, physicochemical, antioxidant, antimicrobial, and PPO‐inhibitory properties. In addition, the applicability of these films for controlling enzymatic browning in fresh‐cut apple slices was investigated. Unlike many previously reported antioxidant‐enriched biopolymer films that focus on individual functional properties, the present study integrates multiple functional evaluations within a single agarose‐based preservation model. Therefore, this work provides a more comprehensive assessment of how matrix composition and functional performance are interconnected in fresh‐cut fruit preservation systems.

## 2 Materials and Methods

### 2.1 Materials

The film was fabricated using agarose. Analytical‐grade agarose (Type I‐A, low electroendosmosis) was obtained from Sigma‐Aldrich (St. Louis, MO, USA). The chemicals used for antioxidant and enzymatic assays were of analytical grade, including 1,1‐diphenyl‐2‐picrylhydrazyl radical (DPPH), ABTS (2,2′‐azino‐bis(3‐ethylbenzothiazoline‐6‐sulfonic acid)), neocuproine (2,9‐dimethyl‐1,10‐phenanthroline), AA, butylated hydroxytoluene (BHT), butylated hydroxyanisole (BHA), α‐tocopherol, catechol, and Folin–Ciocalteu reagent obtained from Sigma‐Aldrich GmbH (Steinheim, Germany). The *Escherichia coli ATCC 25922* and *Staphylococcus aureus ATCC 6538* strains used for antimicrobial tests were purchased from the American Type Culture Collection (ATCC).

### 2.2 AA@Ag Films Preparation

Agarose‐based films were prepared by dissolving agarose in distilled water under continuous stirring and heating until complete solubilization. Glycerol was added as a plasticizer at a concentration of 30% (w/w) relative to the agarose mass. AA was incorporated into the film‐forming solution at concentrations of 0.5%, 1.0%, and 1.5% (w/v) based on the total solution volume, and the mixtures were stirred until homogeneous. The gelatinized modified agarose solution was subjected to vacuum deaeration in a laboratory vacuum desiccator (−0.08 MPa) for approximately 15 min to remove entrapped air bubbles, followed by sonication in an ultrasonic water bath for 30 min to ensure homogeneity of the solution. The deaeration step was performed prior to casting to prevent bubble formation during film drying. The resulting solutions were cast onto Petri dishes (90 mm diameter) to ensure uniform film formation. A constant volume of film‐forming solution was poured into each dish to obtain films with comparable thickness (~80 μm). After drying, the films were carefully peeled and conditioned before further analyses. All film formulations were prepared in at least three independent batches, and all analyses were performed using independently produced films to ensure reproducibility (Fu et al. 2024). No substantial visual or handling‐related differences were observed among independently prepared film batches under the applied preparation conditions, indicating acceptable batch‐to‐batch consistency for the evaluated parameters.

### 2.3 Characterization of AA@Ag Films

#### 2.3.1 Structural Characterization of the Films

The structural and surface properties of the agarose films were characterized by Fourier transform infrared (FTIR) spectroscopy and scanning electron microscopy (SEM), respectively. For FTIR analysis, films were analyzed directly without additional treatment. All measurements were recorded in the spectral region of 4000–1245 cm^−1^ with a resolution of 4 cm^−1^ and 32 scans. The spectra were evaluated using characteristic absorption bands corresponding to hydroxyl (O–H), carbonyl (C=O), C–H, C–O, and glycosidic C–O–C stretching vibrations to assess structural features and possible intermolecular interactions within the film matrix. These spectral features were interpreted primarily in relation to structural organization within the film matrix and were later comparatively discussed alongside the experimentally observed physicochemical behaviors of the films. Microstructural observations were carried out using SEM. Films were also compared before and after enzyme processing. Before imaging, samples were sputter‐coated with gold (Cressington 108 Auto Sputter Coater) to reach a coating thickness of approximately 10 nm to make them conductive, and observations were made at accelerating voltages of 5–20 kV. Changes in surface morphology, microfibrillar density, porosity, and degradation were characterized, and high‐resolution micrographs were captured (Bayrak 2025).

#### 2.3.2 Physical Characterization of the Film

Thickness, moisture content, solubility, swelling degree, and opacity of agarose films were measured using a modified method reported by Rajendran et al. (2024).

##### 2.3.2.1 Thickness

Film thickness was determined using a digital micrometer with an accuracy of ±0.001 mm. For each formulation, measurements were taken from five randomly selected positions on independently prepared flat‐film samples, and the mean value was reported.

##### 2.3.2.2 Moisture Content

To determine moisture content, film samples were initially weighed (*W*_1_) and subsequently dried in a hot‐air oven at 60°C for 24 h. After drying, the final dry mass (*W*_2_) was recorded. Moisture content was calculated according to the following equation:

$$\text{Moisture Content}\left(\%\right)=\frac{W_{1}-W_{2}}{W_{1}}\times 100$$

##### 2.3.2.3 Solubility

Water solubility was evaluated based on the dry matter loss of the films after immersion in distilled water. Initially, samples were dried at 60°C for 24 h to obtain the initial dry mass (*M*_1_). The dried films were then immersed in 20 mL of distilled water under gentle agitation for 1 h. The remaining undissolved fractions were recovered, redried under the same conditions, and weighed again (*M*_2_). Solubility values were calculated using the following equation:

$$\text{Solubility}\left(\%\right)=\frac{M_{1}-M_{2}}{M_{1}}\times 100$$

##### 2.3.2.4 Degree of Swelling

The swelling behavior of the films was analyzed to assess their water uptake capacity. Dried film samples were weighed before immersion and then placed in distilled water. After removing excess surface moisture, the swollen samples were reweighed. Swelling degree (%) was determined using the following equation:

$$\text{Degree of swelling}\left(\%\right)=\frac{W_{2}-W_{1}}{W_{1}}\times 100$$

where *W*_1_ represents the initial dry weight of the film, and *W*_2_ represents the swollen weight after immersion.

##### 2.3.2.5 Opacity

Opacity measurements were performed to evaluate the films' light transmittance. The measurements were obtained by comparing the film's reflectance on white and black backgrounds, using L* values in the CIELAB system. Each film was cut to 4.5 × 1 cm and measured with a colorimeter. During the measurements, the device was calibrated using the manufacturer‐provided white and black reference backgrounds. Opacity was calculated as the ratio of the *L* value measured against a black background to the *L* value measured against a white background, according to the following equation:

$$\text{Opacity}\left(\%\right)=\left(L_{\text{black}}^{*}{/L}_{\text{black}}^{*}\right)\times 100$$

where L* black is the L* (lightness) value of the film measured against the black background, and L* white is the L* (lightness) value of the film measured against the white background.

### 2.4 Determination of Antioxidant Activity

The antioxidant activities of the films were evaluated using a modified version of the method described by Ton‐That et al. For this purpose, 50 mg of agarose‐based film was dispersed in a 50% ethanol solution (ethanol: water, 50:50, v/v), stirred vigorously for 5 h, and then centrifuged at 4000 rpm for 10 min. The resulting supernatant was collected for analysis. All measurements were performed in triplicate, and the results were expressed as radical scavenging capacity (Bayrak and Ozdemir 2025; Ton‐That et al. 2025). The extraction procedure used in this study is designed to recover the soluble antioxidant fraction from the film matrix under controlled conditions. Therefore, the measured antioxidant activity reflects the extractable antioxidant capacity of the films and may provide an indirect indication of the availability of active compounds. However, it should be noted that this approach does not reflect real‐time release behavior under food storage conditions; rather, it simulates the potential release of antioxidant compounds from the film matrix under accelerated extraction conditions.

#### 2.4.1 DPPH Radical Scavenging Activity

DPPH radical scavenging activity was evaluated based on absorbance measurement at 517 nm. For this purpose, a 0.1 mM DPPH stock solution (prepared in methanol) was mixed with the film solution in a 1:3 ratio. The prepared reaction mixtures were incubated in the dark at room temperature for 30 min. At the end of the period, the absorbance was recorded at 517 nm.

#### 2.4.2 ABTS Radical Scavenging Activity

ABTS radical scavenging activity was evaluated spectrophotometrically at 734 nm. The ABTS•^+^ working radical was generated by mixing 7.4 mM ABTS solution with 2.6 mM potassium persulfate, followed by incubation in the dark at room temperature for 12–16 h. For analysis, the film extract and ABTS•^+^ solution were combined at a 1:2 ratio and allowed to react in the dark for 30 min. The decrease in absorbance was subsequently recorded at 734 nm.

#### 2.4.3 Calculation

In both analyses, *A*_0_ represents the absorbance of the radical solution (without film addition); *A*_1_ represents the absorbance of the reaction mixture after film addition. Antioxidant activity was calculated using the following formula:

$$\text{Radical scavenging activity}\;\left(\%\right)=\frac{A_{0}-A_{1}}{A_{0}}\times 100$$

### 2.5 Antibacterial Activity

Two foodborne reference bacterial strains were used for antibacterial assays: *E. coli* *ATCC 25922* (Gram‐negative) and *S. aureus*.

*ATCC 6538* (Gram‐positive). The strains were retrieved from stock cultures and pre‐incubated in Tryptic Soy Broth (TSB) at 37°C for 18–24 h, then diluted with 0.85% NaCl solution to obtain a 0.5 McFarland standard (≈10^8^ CFU/mL). The disk diffusion method was employed to evaluate the direct antimicrobial activity of the films. Disks (5 mm diameter) were aseptically cut from each film type using a sterile punch and placed onto Mueller–Hinton Agar (MHA) plates previously inoculated with the bacterial suspensions. The plates were incubated at 37°C ± 1°C for 18–24 h, after which the inhibition zones formed around each disk were measured in millimeters. Agarose films without AA served as negative controls (Öztürk et al. 2025).

### 2.6 Apple Sample Preparation and Coating

Fresh apples ( *Malus domestica* cv. ‘Tortum’) were obtained at commercial maturity from a local market in Erzurum, Türkiye. This ecotype, native to the microclimate of the Tortum region, was selected for its regional economic importance and distinct enzymatic browning characteristics. Following the principles highlighted by Fanourakis et al. (2025) regarding the influence of the growth environment on shelf life, the samples were strictly screened for quality: only large‐sized fruits (approx. 200 ± 15 g) with uniform color, high firmness, and no mechanical damage were used.

To ensure true biological replication and account for inter‐fruit variability, a large pool of selected apples (*n* > 50) was washed. The apples were then cut into uniform slices (approx. 5 mm thickness). These slices were pooled and randomly assigned to five treatment groups. Prior to the main experiments, preliminary trials were conducted to optimize the coating formulations. Based on these trials, the selected ascorbic acid concentrations (0.5%–1.5%, w/v) were determined to provide the most suitable balance between coating uniformity, structural integrity, and functional performance.

Apple slices were randomly assigned to five treatment groups to ensure a clear experimental design. The treatment groups were as follows:

- agarose solution only (Ag‐1);
- agarose solution containing 0.5% (w/v) ascorbic acid (AA@Ag‐2);
- agarose solution containing 1.0% (w/v) ascorbic acid (AA@Ag‐3);
- agarose solution containing 1.5% (w/v) ascorbic acid (AA@Ag‐4);
- distilled water‐treated samples (control, uncoated).

The AA@Ag‐2 to AA@Ag‐4 formulations represent increasing concentrations of ascorbic acid in the agarose matrix. All samples were coated by immersion in the respective solutions for 1 min.

The product‐to‐solution ratio was set at 1:3, and excess coating solution was allowed to drain after immersion. For each treatment group and sampling day, three independent replicate units were prepared. Each replicate consisted of approximately 25 g of apple slices (approx. 4–5 slices per container) drawn from the randomized pool to ensure that each container represented a distinct experimental unit. The packaged samples were stored in the dark at 4°C ± 1°C for 12 days. Analyses were conducted on Days 0, 1, 3, 5, 7, 9, and 12 throughout the storage period. At each sampling point, three replicate samples were collected; a portion was used for physicochemical determinations (color, PPO activity, and total phenolic content), while the remainder was stored at −18°C for further biochemical analyses. All measurements were performed in triplicate (Ojeda et al. 2019).

### 2.7 Color Analysis

Color measurements of apples coated with agarose and AA@Ag (0.5%–1.5%) films were performed directly on the film‐coated surface using a Minolta CR‐300 colorimeter (Minolta Camera Co., Osaka, Japan). Measurements were conducted under D65 illumination and a 0° standard observer angle, with a 0.8 cm aperture in reflectance mode. Before analysis, the instrument was calibrated using a white ceramic reference plate (CR‐A43) supplied by the manufacturer (Ramirez et al. 2015). For each treatment, three independent 25 g packages of apple slices were used as experimental units (*n* = 3). Color measurements were performed directly on the slices within each package rather than on whole fruits. To obtain representative L* (lightness), a* (redness/greenness), and b* (yellowness/blueness) values for each replicate, three different points were randomly selected on the surface of the slices within each package, and the average of these measurements was used for statistical analysis. In addition, the browning index (BI) was calculated using the following equation (Holderbaum et al. 2010):

$$\mathrm{BI}=\left[100\left(x-0.31\right)\right]/0.172$$

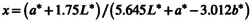




### 2.8 Determination of PPO Inhibitory Activity Using AA@Ag and Ag‐1 Films

Samples were collected at predetermined intervals during storage (Days 0–12) to determine PPO activity in apple slices coated with AA@Ag and agarose films.

PPO activity was evaluated separately for each treatment group (control, Ag‐1, AA@Ag‐2, AA@Ag‐3, and AA@Ag‐4) to allow direct comparison of coating effects.

Before analysis, the coating layer on the apple surface was carefully and consistently removed using a sterile spatula to expose the underlying apple tissue. This procedure was performed by the same operator across all replicates to ensure methodological consistency. Special care was taken to minimize mechanical stress during removal in order to avoid potential alterations in enzymatic activity. The superficial tissue layer (approximately 2 mm) was then sampled for enzyme extraction. Approximately 1 g of apple from each sample was homogenized in 10 mL of 0.1 M phosphate buffer (pH 6.5) at 4°C. The homogenate was centrifuged at 10,000 × ***g*** for 15 min at 4°C, and the supernatant was collected. This supernatant served as the crude enzyme extract for determining PPO activity (Bayrak and Omeroglu 2026). PPO activity was measured spectrophotometrically using catechol as the substrate. Absorbance was recorded at 420 nm, and one unit of enzyme activity was defined as the amount of enzyme causing a 0.001 change in absorbance per minute (Öztürk 2025).

This approach was adopted to assess PPO activity in apple tissue itself while minimizing direct interference from coating components (e.g., AA) in in vitro enzyme analysis.

### 2.9 Statistical Analysis

All tests were repeated three times, and results were presented as mean ± standard deviation (SD). For the analysis of physicochemical and enzymatic parameters, treatment (film type) and storage time were considered as fixed factors in a two‐way ANOVA model. Interaction effects between treatment and storage time were also evaluated. Prior to analysis, normality and homogeneity of variance were assessed using the Shapiro–Wilk and Levene tests, respectively. When significant differences were detected, Tukey's HSD post hoc test was applied. Storage time was treated as an independent factor rather than a repeated measure due to destructive sampling at each time point. Statistical significance was accepted at *p* < 0.05. Statistical analysis was performed using GraphPad Prism 8.0 (GraphPad Software, San Diego, CA, USA) calculation software.

## 3 Results and Discussion

### 3.1 Preparation of AA@Ag Films

**FIGURE 1:**
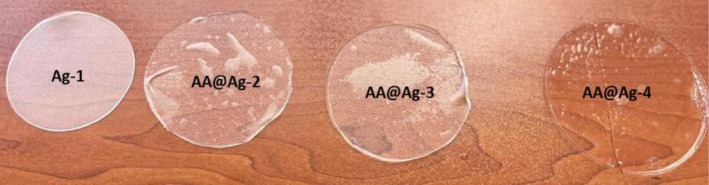
Prepared Agar‐1 (0%), AA@Ag‐2 (0.5%), AA@Ag‐3 (1.0%), AA@Ag‐4 (1.5%) films.

The gel strength of agarose may be insufficient to satisfy the performance requirements of certain applications. Therefore, modification of agarose‐based systems is often explored to improve their functional properties. In this context, the present study aims to develop agarose as an active packaging material with tailored functional characteristics. Agarose films at a concentration of 1.0% (w/w) were prepared as follows: predetermined amounts of agarose and AA at varying concentrations (0.5%–1.5%, w/v) were added to distilled water, and the mixture was heated to the boiling point to ensure complete dissolution of the polymer (Table 1, Figure 1). The resulting solution was then cast into Petri dishes under controlled conditions and allowed to cool at room temperature, forming films.

**TABLE 1:** Formulations of AA@Ag films with different AA concentrations.

Films	Agoros (%)	AA (%)
Ag‐1	1.0	0.0
AA@Ag‐2	1.0	0.5
AA@Ag‐3	1.0	1.0
AA@Ag‐4	1.0	1.5

Agarose is a hydrophilic polysaccharide capable of readily forming gels. It dissolves upon heating and, upon cooling, forms a gel structure.

This thermoreversible behavior enables the formation of continuous and uniform film matrices suitable for coating applications (Xiao et al. 2019). There are similar studies in the literature. For instance, Fu et al. modified agarose films with p‐hydroxybenzoic acid (Ph) and 3,4‐dihydroxybenzoic acid (Da) and applied them as coatings for fish products. In another study, the same research group developed gallic acid–modified agarose films to extend the shelf life of grass carp (*Ctenopharyngodon idellus*; Fu et al. 2024). Similarly, Yu et al. fabricated agarose‐based films incorporating purple sweet potato extract and polyvinyl alcohol to maintain the freshness of meat products (Yu et al. 2023).

### 3.2 Characterization of the Prepared Films Using Different Methods

#### 3.2.1 Structural Characterization of the Films

##### 3.2.1.1 FT‐IR Spectrum

**FIGURE 2:**
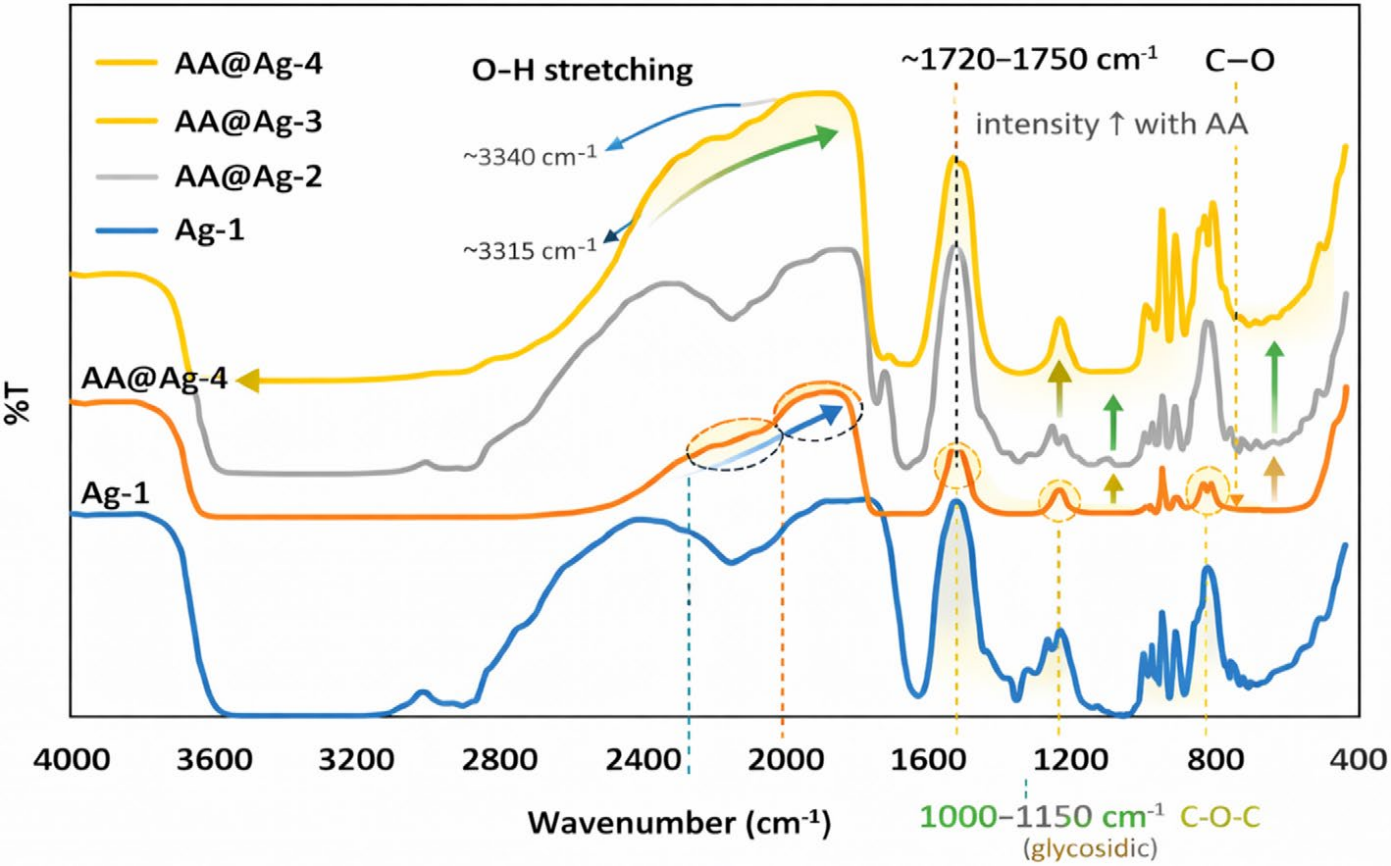
FT‐IR spectra of agarose films containing Ag‐1 and different concentrations of AA (AA@Ag‐2, AA@Ag‐3, AA@Ag‐4). Changes in the O–H, C=O, and C–O–C regions indicate the interaction of AA with the film matrix.

FT‐IR analyses were used to evaluate molecular interactions upon addition of AA to the agarose‐based film matrix. In the Ag‐1 (pure agarose) film, characteristic bands specific to the polysaccharide structure were observed: a broad band corresponding to O–H stretching vibrations at approximately 3300–3400 cm^−1^, C–H stretching around 2920–2930 cm^−1^, a deformation band corresponding to bound water in the region of 1630–1640 cm^−1^, and characteristic absorptions of C–O–C glycosidic bonds in the range of 1000–1150 cm^−1^ (Figure 2).

Compared with neat agarose films, AA‐containing formulations exhibited noticeable spectral alterations in several characteristic regions. The O–H stretching band broadened and shifted slightly, indicating changes in the hydrogen‐bonding environment within the polymer network. In addition, increased absorption intensity around 1720–1750 cm^−1^ was associated with carbonyl‐containing structures introduced by AA. Variations observed within the glycosidic C–O–C region (1000–1200 cm^−1^) further suggested that AA incorporation affected the local structural organization of the agarose matrix. These spectral variations became progressively more evident as AA concentration increased. These observations are consistent with previous studies. Similar spectral changes in O–H and C=O regions have been reported following the incorporation of ascorbate derivatives into polysaccharide‐based systems. In addition, FT‐IR studies on polysaccharides have demonstrated that variations in C–O–C stretching vibrations (1000–1200 cm^−1^) are sensitive to structural organization and local interactions within polymer matrices (Manna et al. 2025; Onofre‐Cordeiro et al. 2018; Zhu et al. 2011).

##### 3.2.1.2 SEM Analysis

**FIGURE 3:**
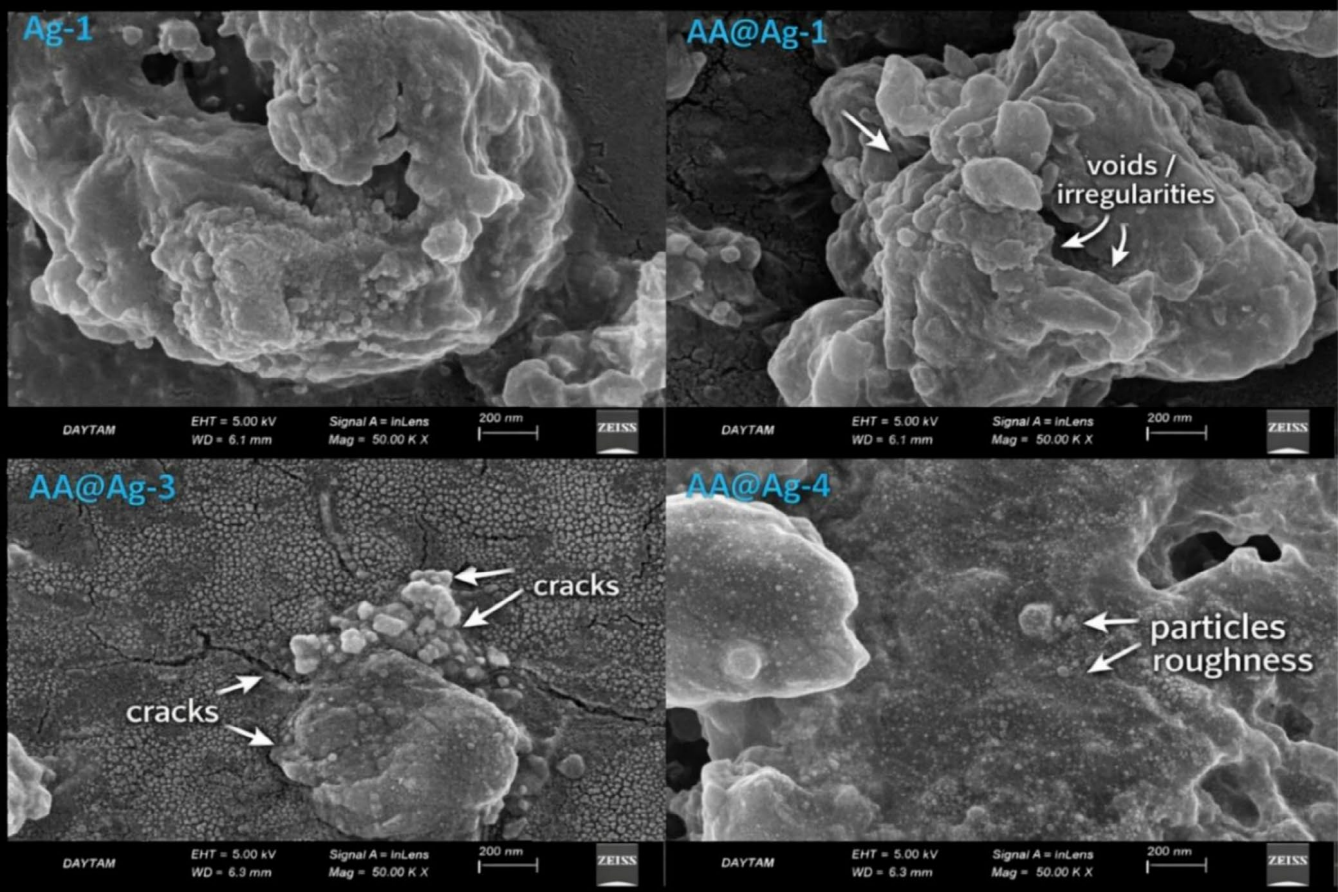
SEM images of agarose‐based films at 50,000× magnification: Ag‐1, AA@Ag‐2, AA@Ag‐3, and AA@Ag‐4. Arrows indicate cracks, voids, and particle formations. Scale bar: 200 nm.

SEM analyses revealed pronounced alterations in the surface morphology of agarose (Ag‐1) films and those containing varying concentrations of AA (AA@Ag‐2, AA@Ag‐3, AA@Ag‐4) (Figure 3). As illustrated in Figure 3, the pure agarose film (Ag‐1) exhibited a relatively compact and continuous surface with localized irregular domains at the nanoscale. Following AA incorporation, distinct concentration‐dependent morphological changes became evident. The film with a low concentration of AA (AA@Ag‐2) exhibited discrete surface domains and localized discontinuities, which may indicate a partial and non‐uniform distribution of AA within the polymer matrix. At an intermediate concentration (AA@Ag‐3), a highly granular, densely packed surface structure was observed, suggesting increased aggregation and heterogeneous dispersion of AA‐related domains within the film network. At the highest concentration (AA@Ag‐4), the surface appeared more continuous overall, with fine particulate structures distributed more uniformly across the matrix, indicating improved AA dispersion at the nanoscale.

Previous studies have similarly shown that incorporating low‐molecular‐weight additives into polysaccharide matrices can alter aggregation behavior, phase distribution, and surface organization in a concentration‐dependent manner. Therefore, the morphological differences observed in the present study should be interpreted primarily in terms of structural organization rather than as direct evidence of functional enhancement (Manna et al. 2025; Martínez et al. 2023; Shin et al. 2022; Wang et al. 2011). The structure‐related functional interpretations derived from FTIR and SEM analyses were evaluated within the scope of the observed physicochemical findings; however, complementary permeability and mechanical analyses would provide a more comprehensive understanding of these relationships.

#### 3.2.2 Physical Characterization of the Film

The addition of AA resulted in significant changes in the films' physical properties. Thickness values were similar between groups (≈80–81 μm), and the addition of AA did not create a significant difference (*p* > 0.05). However, moisture content decreased from 8.03% in the Ag‐1 control film to 6.70% in the AA@Ag films, which can be explained by AA forming hydrogen bonds with the agarose matrix, thereby reducing its free water‐holding capacity (*p* < 0.05). Similarly, solubility decreased from 25.06% in the control film to 18.33%–20.53% in the AA‐doped films, indicating that the matrix became more resistant to water (*p* < 0.05; Table 2).

**TABLE 2:** Thickness, moisture content, solubility, swelling, and opacity values of Ag‐1 and AA‐enriched agarose (AA@Ag) films (mean ± SD, *n* = 3). Different lowercase letters (a–c) within the same column indicate statistically significant differences (*p* < 0.05, Tukey's HSD test).

Sample	Thickness ± SD (μm)	Moisture content %	Solubility %	Swelling degree %	Opacity %
Ag‐1	80.53 ± 0.503^a^	8.03 ± 0.057^a^	25.06 ± 2.676^a^	44.36 ± 2.259^a^	44.96 ± 0.321^c^
AA@Ag‐2	80.86 ± 0.230^a^	7.00 ± 0.099^b^	20.53 ± 1.350^b^	39.73 ± 0.472^b^	48.40 ± 1.113^b^
AA@Ag‐3	80.83 ± 0.208^a^	6.84 ± 0.052^bc^	19.13 ± 0.680^bc^	39.56 ± 0.351^b^	50.33 ± 0.808^a^
AA@Ag‐4	80.80 ± 0.173^a^	6.70 ± 0.178^c^	18.33 ± 0.737^c^	39.40 ± 0.199^b^	50.96 ± 0.680^a^

The swelling ratio decreased from 44.36% in the control film to 39.40%–39.73% in AA@Ag films, indicating the formation of a more compact polymer network (*p* < 0.05; Table 2). Opacity increased from 44.96% to 50.96% with increasing AA concentration, attributed to enhanced light scattering resulting from structural modifications within the film matrix (Table 2). This characteristic may be advantageous for protecting light‐sensitive foods; however, consumer acceptance necessitates an optimal balance between transparency and opacity. While reductions in moisture content, solubility, and swelling indicate improved structural integrity, it should also be considered that increased matrix density can affect mass‐transfer properties, including the diffusion and release of active compounds. Therefore, these structural changes can present both advantages and potential limitations depending on the intended application. The present findings are consistent with previous reports demonstrating that incorporating active compounds or nanocomposite additives induces comparable alterations in film properties (Hosseini et al. 2021; Nisar et al. 2018; Sani et al. 2019; Suput et al. 2016). In particular, reductions in solubility and swelling ratio represent important improvements for preserving mechanical integrity and extending shelf‐life performance. Similarly, increased opacity values reported by Ramirez et al. and Rajendran et al. were positively associated with enhanced light‐barrier functionality in food packaging systems (Rajendran et al. 2024; Ramirez et al. 2015).

### 3.3 Antioxidant Activity

One of the major challenges in the food industry is the limited shelf life of products, resulting from oxidative reactions such as enzymatic browning and oxidative degradation (Soliva‐Fortuny and Martin‐Belloso 2003). To minimize oxidative deterioration in food systems, increasing attention has been paid to incorporating antioxidant compounds into edible films and coatings. Such approaches may improve the functional performance of biopolymer‐based systems by reducing oxidative reactions under model conditions (Sánchez‐González et al. 2011). Antioxidants, regardless of their specific mechanism of action, are known to reduce oxidative damage in model systems by scavenging free radicals (Pokorný 2007). Consequently, recent research has increasingly focused on integrating plant extracts (Akhtar et al. 2012; Zeng et al. 2013), essential oils (Blanco‐Fernandez et al. 2013; Bonilla et al. 2013; Perdones et al. 2014), and natural antioxidants such as α‐tocopherol into biopolymer‐based films.

**FIGURE 4:**
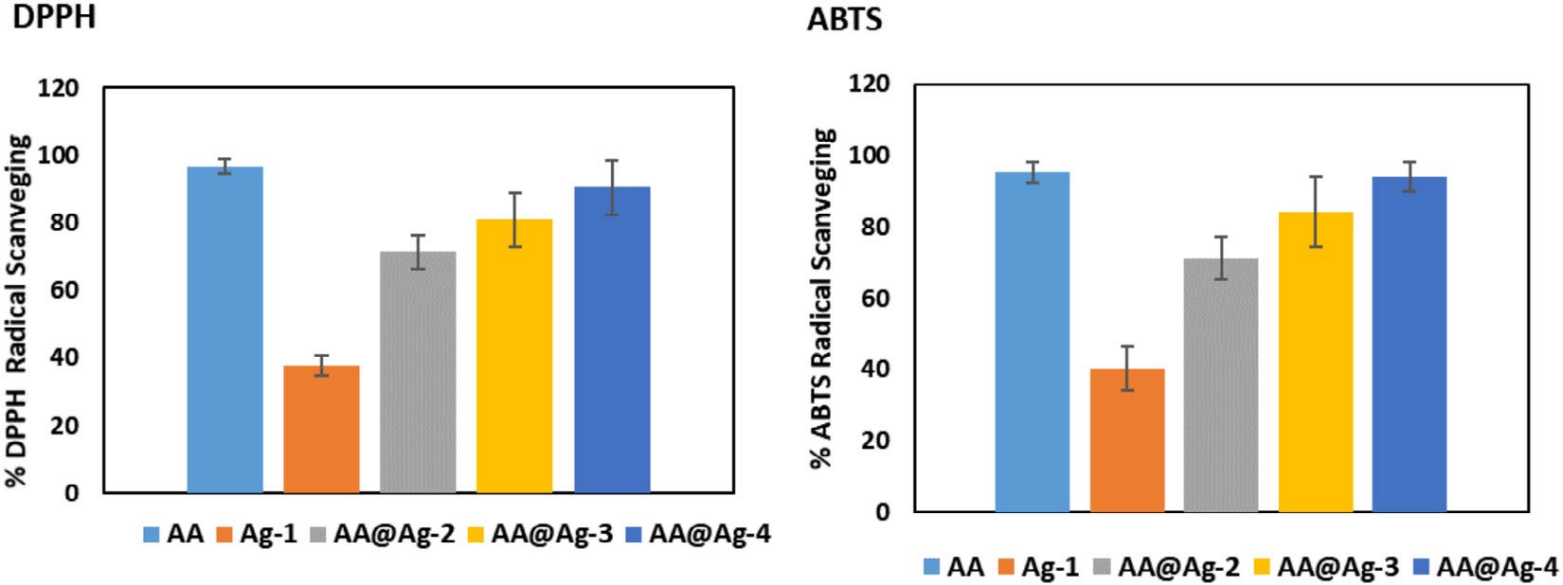
DPPH and ABTS radical scavenging activities of AA, Ag‐1, and AA@Ag (2–4) samples. Results are given as mean ± standard deviation (*n* = 3).

In the present study, the antioxidant activities of agarose‐based films, a naturally derived biopolymer matrix, enriched with different concentrations of AA (AA@Ag), were evaluated using DPPH and ABTS radical scavenging assays (Table 3, Figure 4).

**TABLE 3:** Antioxidant activity of standard AA, Ag‐1, and AA‐enriched AA@Ag (2–4) films (mean ± SD, *n* = 3). Different lowercase letters (a–d) within the same column indicate statistically significant differences (*p* < 0.05).

Films/standart	DPPH radical giderme (RSE %)	ABTS radical giderme (RSE %)
AA*	96.60 ± 2.000^a^	95.30 ± 3.764^a^
Ag‐1	37.76 ± 3.800^d^	40.33 ± 6.493^c^
AA@Ag‐2	71.33 ± 5.132^c^	71.20 ± 6.366^b^
AA@Ag‐3	80.86 ± 8.800^bc^	84.20 ± 10.790^ab^
AA@Ag‐4	90.53 ± 8.053^ab^	94.03 ± 4.874^a^

* The results were compared with standard antioxidant AA (*p* < 0.05).

According to the findings, the standard AA showed the highest free radical scavenging activity (DPPH: 96.60% ± 2.00%; ABTS: 95.30% ± 3.76%). In contrast, the Ag‐1 film had a very low antioxidant capacity (DPPH: 37.76% ± 3.80%; ABTS: 40.33% ± 6.49%). However, the addition of AA significantly increased the film's antioxidant capacity, depending on concentration (*p* < 0.05). For example, in the AA@Ag‐2 sample, DPPH and ABTS efficiencies were 71.33% ± 5.13% and 71.20% ± 6.37%, respectively, while in the AA@Ag‐4 sample, these efficiencies were 90.53% ± 8.05% and 94.03% ± 4.87%, respectively. One‐way ANOVA and Tukey HSD tests showed that the addition of AA significantly increased antioxidant activity (*p* < 0.05).

The enhanced radical‐scavenging activity observed in AA‐containing films can be associated with the redox‐active structure of AA. Due to its electron‐donating capability, AA can interact with reactive radical species and reduce oxidative chain propagation in model systems (Tošović et al. 2017; Zhao et al. 2020). In contrast, agarose itself exhibits relatively limited antioxidant behavior because its polysaccharide backbone lacks highly reactive antioxidant moieties (Fu et al. 2024; He et al. 2022). It should also be considered that the antioxidant activities reported here were obtained under extraction‐based assay conditions and therefore do not directly represent the real‐time release behavior or in situ antioxidant performance of the films during food storage. Accordingly, these results should be interpreted as comparative indicators of antioxidant potential rather than direct evidence of food preservation performance. In this context, agarose‐AA composite films may delay oxidative changes in food systems; however, further studies under real food storage conditions are required to better evaluate their practical effectiveness. Notably, AA@Ag films containing higher AA concentrations exhibited antioxidant capacities approaching those of pure AA under extraction conditions, suggesting increased availability of active compounds within the film matrix rather than confirming in situ functionality. Future studies focusing on release kinetics and real‐time antioxidant behavior under food storage conditions would further clarify the functional performance of these films in practical preservation systems.

### 3.4 Antibacterial Activity

Microbial contamination remains a major challenge affecting both the microbiological safety and storage stability of food products. Therefore, enhancing the antimicrobial performance of food packaging materials has become a major research focus within the field of active packaging (Ahmed et al. 2022; Öztürk et al. 2026; Sofi et al. 2018).

**FIGURE 5:**
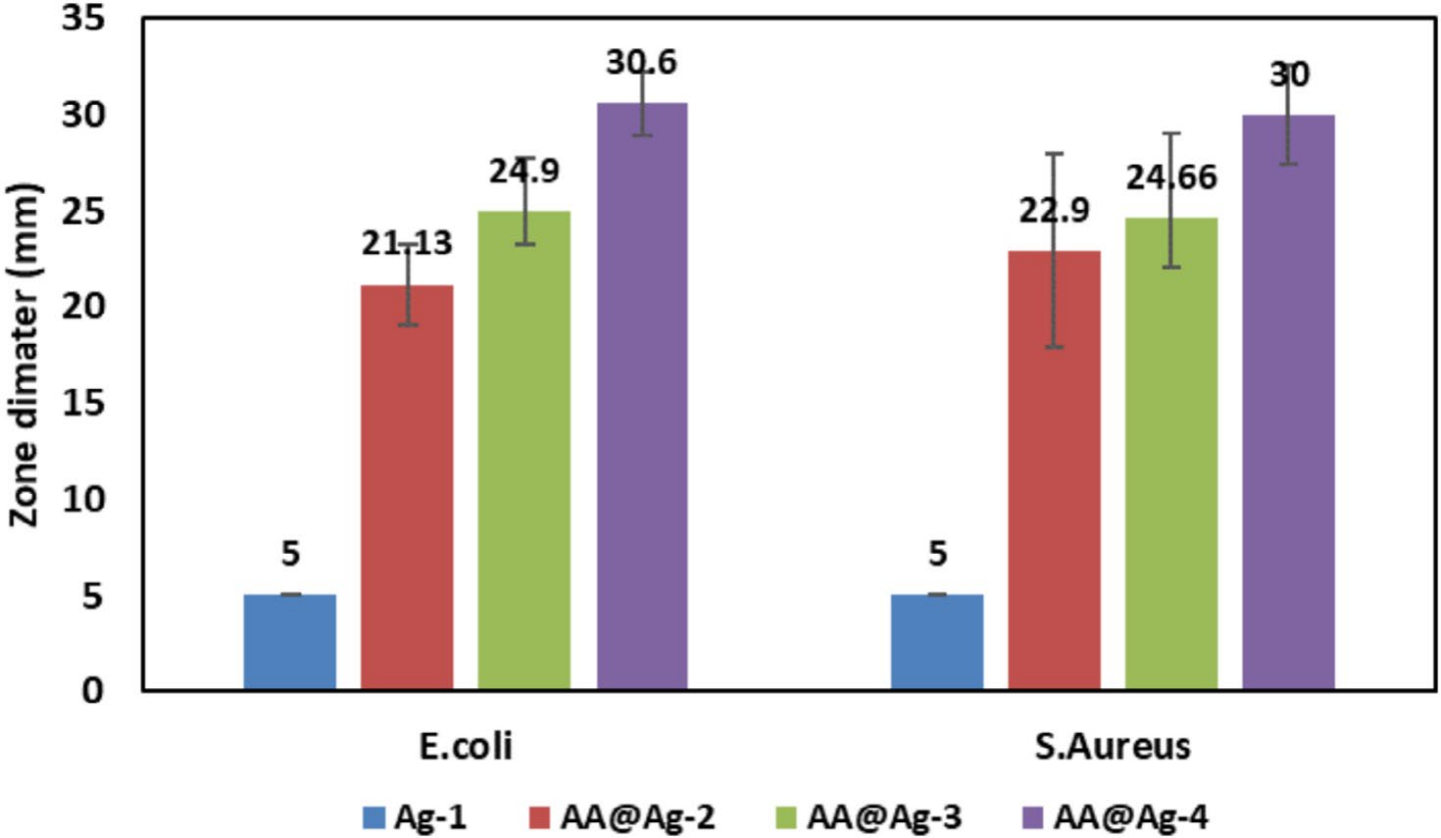
The antimicrobial effects of AA@Ag and agarose films against *E. coli* and *S. aureus* ; inhibition zone diameters (total diameter including the disk, mm; mean ± SD; *n* = 3).

In the present study, the antimicrobial activities of Ag‐1, AA@Ag‐2, AA@Ag‐3, and AA@Ag‐4 films against *E. coli* and *S. aureus* were evaluated using the disk diffusion assay (Figure 5). No inhibition zone was detected for the control Ag‐1 film; the measured value of 5 mm corresponded solely to the disk diameter and therefore did not indicate genuine antimicrobial activity. In contrast, inhibition zone diameters increased progressively with AA concentration in AA@Ag films. Notably, the AA@Ag‐4 film exhibited the strongest inhibition against both *E. coli* (30.6 mm) and *S. aureus* (30.0 mm), indicating that increasing AA concentration is associated with larger inhibition zones under agar diffusion conditions (Figure 5).

Statistical analysis demonstrated that differences between AA‐containing films and both Ag‐1 and control groups were significant (*p* < 0.05). Moreover, AA@Ag‐4 produced significantly larger inhibition zones than AA@Ag‐2 and AA@Ag‐3 for both bacterial strains (*p* < 0.05). Collectively, these findings indicate that the presence and concentration of AA influence the inhibition zone diameters observed in the agar diffusion assay.

It should be noted that the disk diffusion method provides only a preliminary and indirect indication of antimicrobial potential, as the observed inhibition zones are strongly influenced by the diffusion behavior of active compounds within the agar medium. Within this context, the results obtained in this study should be interpreted as evidence of antimicrobial potential under in vitro conditions, rather than direct proof of effectiveness in practical food applications. The results obtained in this study are consistent with previous reports indicating that AA‐containing systems can exhibit antibacterial activity under in vitro conditions (Hernandez‐Patlan et al. 2018; Mukhopadhyay et al. 2016; Taşdemir et al. 2022). Nevertheless, further in situ food microbiology studies under real storage conditions are required to validate the antimicrobial effectiveness of these films in practical food preservation systems.

### 3.5 Color and Browning Analysis

Enzymatic browning is a major postharvest quality problem in fresh produce and is primarily associated with PPO‐catalyzed oxidation reactions occurring after tissue disruption. In apple tissues rich in phenolic substrates, these reactions promote the formation of dark‐colored pigments that contribute to deterioration in visual quality (Christopoulos and Tsantili 2011). In this context, it is important to distinguish direct antibrowning performance from broader preservation outcomes. While reductions in browning index, maintenance of L values, and lower PPO activity provide evidence of antibrowning efficacy under the tested conditions, broader implications, such as shelf‐life extension or commercial applicability, depend on additional microbiological, physicochemical, and sensory factors that were not comprehensively evaluated in the present study.

**FIGURE 6:**
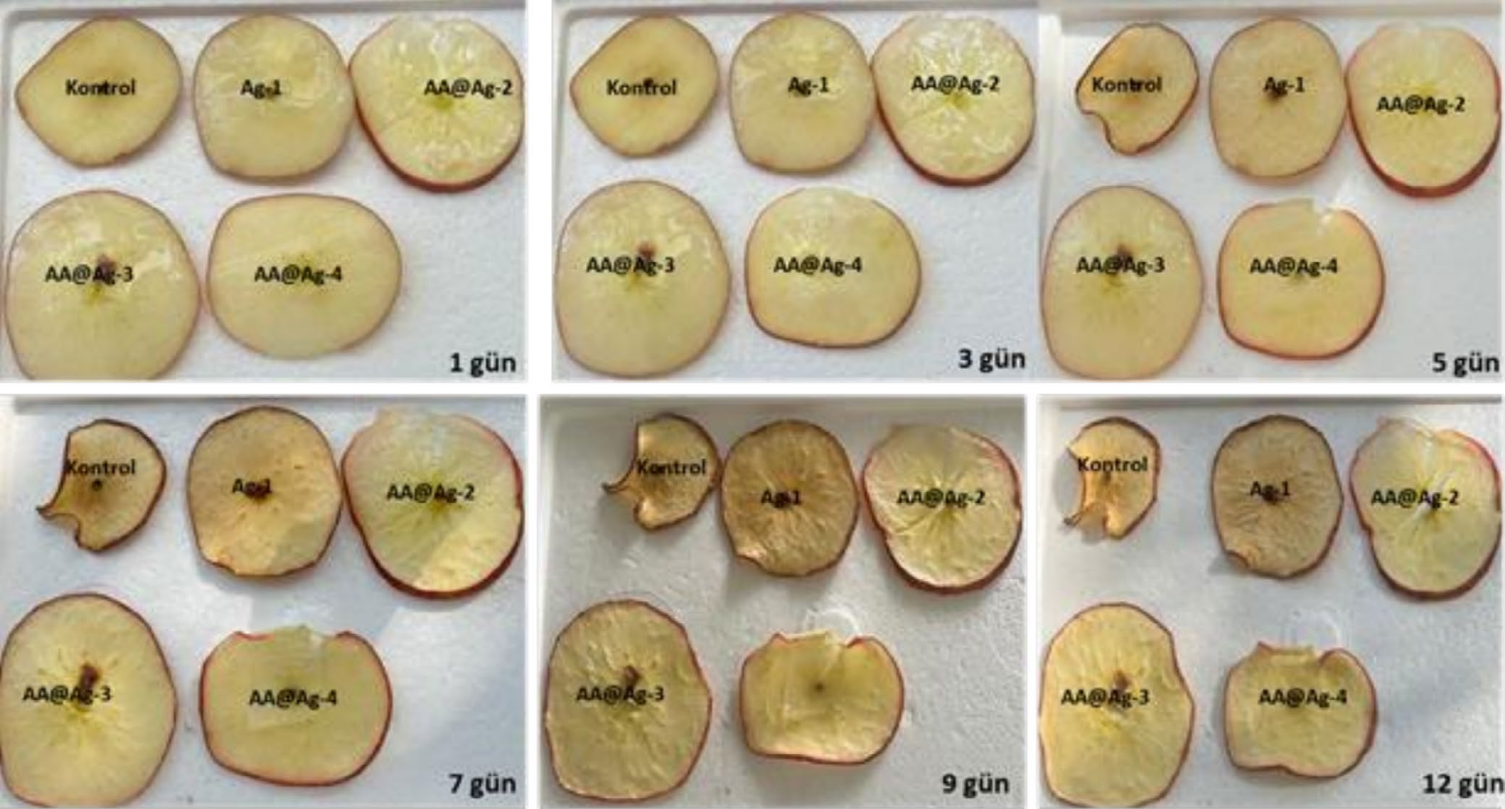
Changes in the visual appearance of apples coated with the control group, pure Ag‐1 film, and films containing different concentrations of AA [AA@Ag‐2 (0.5% AA), AA@Ag‐3 (1.0% AA), and AA@Ag‐4 (1.5% AA)] during 12 days of storage.

As illustrated in Figure 6, agarose coatings enriched with AA (AA@Ag) delayed the progression of enzymatic browning in fresh‐cut apple slices under the experimental conditions applied in this study. After 12 days of storage, apple slices treated with AA@Ag‐2, AA@Ag‐3, and AA@Ag‐4 maintained substantially higher color stability, whereas visible browning appeared as early as Day 5 in both the control and Ag‐1 groups. This protective effect may be associated with the oxygen‐scavenging and reducing properties of AA within the film matrix, which can influence PPO‐mediated reactions and quinone formation processes (Ali et al. 2015). However, it should also be considered that browning behavior is influenced by intrinsic fruit characteristics, including cultivar‐specific properties and maturity level, which were, to some extent, controlled in this study through sample selection.

The results presented in Table 4 further corroborate these observations. The decline in L* values in uncoated apples during storage, accompanied by concurrent increases in a* and BI values, clearly indicates progressive browning and deterioration of visual quality. However, samples coated with Ag‐1 exhibited slightly lower BI values than the control; coatings supplemented with AA, particularly at 1.0% and 1.5%, significantly suppressed browning (*p* < 0.05). Notably, by Day 12, the BI value of apples coated with AA@Ag‐1.5% was 27.5, whereas the control reached 41.5 (*p* < 0.05). This trend suggests that the presence of AA may contribute to reducing PPO‐related oxidative changes in apple slices during storage under the tested conditions. In addition, the use of a single ecotype (cv. ‘Tortum’) and controlled selection criteria may have reduced variability in browning responses among samples. Comparable findings have been reported in previous studies: Habibie et al. and Zhou et al. observed higher L* retention and reduced browning in walnut kernels coated with AA and walnut green husk extract; similarly, Zhao et al. reported reduced browning in fresh‐cut potatoes treated with methyl jasmonate, while Xiong et al. documented analogous effects in apples treated with quercetin combined with antioxidant systems (Habibie et al. 2019; Xiong et al. 2025; Zhao et al. 2020; Zhou FuHui et al. 2019).

**TABLE 4:** Color parameters (L*, a*, b*) and browning index (BI) of fresh‐cut apples coated with agarose and AA@Ag films during storage (mean ± SD, *n* = 3). Different lowercase letters within the same column for each storage day indicate statistically significant differences (*p* < 0.05, Tukey's HSD test).

Day	Group	L*	a*	b*	BI
1	Control	78.2 ± 0.5^a^	−1.2 ± 0.2^a^	18.5 ± 0.6^a^	12.3 ± 0.7^a^
	Ag‐1	78.0 ± 0.7^a^	−1.3 ± 0.3^a^	18.3 ± 0.4^a^	12.1 ± 0.8^a^
	AA@Ag‐2	78.4 ± 0.6^a^	−1.1 ± 0.2^a^	18.4 ± 0.5^a^	12.0 ± 0.6^a^
	AA@Ag‐3	78.6 ± 0.4^a^	−1.0 ± 0.2^a^	18.7 ± 0.3^a^	11.9 ± 0.5^a^
	AA@Ag‐4	78.8 ± 0.5^a^	−1.0 ± 0.2^a^	18.8 ± 0.5^a^	11.8 ± 0.6^a^
12	Control	65.5 ± 0.8^e^	5.8 ± 0.5^a^	21.2 ± 0.6^a^	41.5 ± 1.2^a^
	Ag‐1	67.2 ± 0.9^d^	5.0 ± 0.4^b^	21.0 ± 0.5^a^	38.9 ± 1.1^b^
	AA@Ag‐2	70.4 ± 0.7^c^	4.0 ± 0.3^c^	20.6 ± 0.6^ab^	33.5 ± 1.0^c^
	AA@Ag‐3	72.8 ± 0.8^b^	3.2 ± 0.4^d^	20.1 ± 0.5^b^	29.8 ± 1.2^d^
	AA@Ag‐4	74.0 ± 0.6^a^	2.8 ± 0.3^d^	19.8 ± 0.4^b^	27.5 ± 1.0^e^

### 3.6 PPO Inhibitory Activity of AA@Ag and Ag‐1 Films

Enzymatic browning in fruits and vegetables begins with the oxidation of phenolic compounds to quinones by PPO as a result of stresses such as tissue cutting, oxygen exposure, or post‐harvest processing; these quinones then polymerize into brown melanin‐like pigments, and this color change negatively affects the appearance, nutritional quality, and perception of the product (Aksoy 2020). PPO is a copper‐containing oxidoreductase enzyme that oxidizes phenols to quinones as a substrate, thus forming the catalytic component of the browning reaction (Öztürk et al. 2022; Sui et al. 2023).

The data presented in Table 5 demonstrate pronounced temporal changes in PPO activity in apples treated with control (film‐free), Ag‐1, and ascorbic‐acid‐containing films (AA@Ag‐2, AA@Ag‐3, AA@Ag‐4) during 12 days of storage. In the control group, enzyme activity increased markedly from approximately 50 U/mL at the initial stage to 131.30 U/mL on Day 12. A comparable trend was observed in the Ag‐1 film group, where activity reached 115.50 U/mL, indicating that the agarose matrix alone exerted minimal inhibitory influence on enzymatic oxidation. In contrast, films enriched with AA significantly suppressed PPO activity, yielding Day 12 values of 81.33, 80.83, and 79.20 U/mL for AA@Ag‐2, AA@Ag‐3, and AA@Ag‐4, respectively. Statistical analysis confirmed that all AA‐containing films produced significantly lower enzyme activity than both the control and Ag‐1 treatments (*p* < 0.05), indicating that AA‐containing films are associated with lower PPO activity under the experimental conditions applied in this study.

**TABLE 5:** PPO activity (U/mL) of fresh‐cut apples coated with agarose and AA@Ag films during storage (mean ± SD, *n* = 3). Different superscript letters within the same row indicate statistically significant differences among treatments (*p* < 0.05, Tukey's HSD test).

Day	Control	Ag‐1	AA@Ag‐2	AA@Ag‐3	AA@Ag‐4
1	50.00 ± 1.00^a^	51.20 ± 1.31^a^	51.20 ± 1.31^a^	50.63 ± 0.55^a^	50.73 ± 0.64^a^
3	64.33 ± 4.04^a^	55.73 ± 4.02^b^	55.23 ± 3.25^b^	53.73 ± 1.61^b^	55.60 ± 1.83^b^
5	87.00 ± 6.24^a^	64.86 ± 3.25^b^	62.33 ± 1.53^bc^	60.23 ± 2.07^c^	61.43 ± 0.76^bc^
7	103.50 ± 5.46^a^	86.43 ± 5.29^b^	67.50 ± 2.40^c^	66.96 ± 1.51^c^	67.26 ± 2.01^c^
9	117.00 ± 16.04^a^	106.00 ± 10.13^ab^	75.53 ± 4.75^c^	74.23 ± 3.80^c^	73.96 ± 2.32^c^
12	131.30 ± 9.24^a^	115.50 ± 5.55^b^	81.33 ± 1.35^c^	80.83 ± 1.53^c^	79.20 ± 3.86^c^

It should be noted that PPO activity and browning susceptibility are strongly influenced by cultivar‐dependent characteristics and preharvest conditions. In the present study, the use of a single ecotype (cv. ‘Tortum’) and selection for uniform maturity and physical quality helped reduce variability. Although the observed reduction in PPO activity is consistent with the antibrowning effect of AA, the underlying mechanism cannot be conclusively determined based on the present data. The decrease in enzyme activity may be associated with multiple factors, including oxygen limitation, substrate availability, reduction of o‐quinones, possible direct enzyme inhibition, or changes in tissue physiology induced by the coating. However, the observed responses should not be attributed exclusively to the coating system, as intrinsic fruit characteristics, tissue physiology, storage conditions, and matrix–food interactions may also contribute to PPO behavior and the development of browning. Previous studies have suggested that AA can participate in redox reactions, such as the reduction of o‐quinones back to diphenols, and may also influence oxidative processes in plant tissues (Bradshaw et al. 2011; Cárdenas‐Moreno et al. 2023; Flores‐Sauceda et al. 2025; Lo'Ay and Dawood 2017). However, these mechanisms should be considered as possible explanations rather than definitive interpretations in the context of the present study.

Systems incorporating essential oils, phenolic extracts, or AA have been associated with reduced enzymatic browning under in vitro or model conditions (Ali et al. 2025).

Accordingly, the present results should be interpreted as evidence of PPO modulation in apple slices under the experimental conditions applied, rather than as definitive proof of a specific inhibitory mechanism or of generalized applicability across different food systems (Ma et al. 2024).

In addition to these considerations, the transferability and practical applicability of the observed effects should be interpreted with caution. While the present study provides evidence of PPO modulation in fresh‐cut apple slices ( *Malus domestica* cv. ‘Tortum’), the extent to which similar responses would be observed in other apple cultivars remains uncertain due to known differences in phenolic composition, PPO activity, and tissue structure. This uncertainty is expected to be even greater across different fruit and vegetable systems, where physiological and biochemical characteristics may significantly influence the response to coating treatments (Soliva‐Fortuny and Martin‐Belloso 2003). Furthermore, from a practical perspective, factors such as coating uniformity at scale, solution stability during processing, cost‐effectiveness relative to conventional antibrowning methods, compatibility with existing processing lines, and regulatory considerations must be addressed. Although the reductions in PPO activity and associated browning parameters are notable under laboratory conditions, their direct impact on consumer perception and commercial performance requires further validation through sensory and industrial‐scale studies.

## 4 Conclusion

Enzymatic browning of apple slices represents a major technological challenge in the food industry, as it directly compromises product freshness and visual quality. In this study, agarose‐based biopolymer films functionally enriched with AA were investigated as active packaging materials through integrated structural, physicochemical, and biological analyses. FT‐IR and SEM results confirmed the effective incorporation of AA into the polymer matrix, indicating intermolecular hydrogen bonding and the formation of homogeneous surface morphologies under the tested conditions. Functional assays demonstrated that the films exhibit in vitro antioxidant and antimicrobial activities, both of which increased in a concentration‐dependent manner upon incorporation of AA. Application trials on fresh‐cut apple slices (cv. ‘Tortum’) validated the coatings' performance under laboratory conditions, demonstrating a notable inhibition of enzymatic browning and color degradation during storage. However, the simultaneous control of oxidative and microbial spoilage in commercial‐scale fresh‐cut systems, as well as the transferability of these findings to other fruit and vegetable species with different physiological characteristics, remains to be demonstrated. The findings highlight the technological potential of integrating natural antioxidant systems into sustainable food‐packaging research, providing a basis for future studies on large‐scale feasibility and commercial applicability.

## Author Contributions

**Hasan Ozdemir:** visualization, resources, data curation, methodology. **Songul Bayrak:** conceptualization, methodology, software, data curation, formal analysis, investigation, validation, writing – original draft, writing – review and editing.

## Funding

This study was supported by the Atatürk University Scientific Research Projects Coordination Unit (Project No. FBA‐2024‐14460), which provided support for the materials, consumables, and instrumentation used in this study. Open access funding provided by the Scientific and Technological Research Council of Türkiye (TÜBİTAK).

## Ethics Statement

The authors have nothing to report.

## Conflicts of Interest

The authors declare no conflicts of interest.

## Data Availability

The data supporting the findings of this study are available from the corresponding authors upon reasonable request.
